# A virtual computer lab for distance biomedical technology education

**DOI:** 10.1186/1472-6920-8-12

**Published:** 2008-03-13

**Authors:** Craig Locatis, Anibal Vega, Medha Bhagwat, Wei-Li Liu, Jose Conde

**Affiliations:** 1 Office of High Performance Computing and Communications, National Library of Medicine, Bethesda, Maryland, USA; 2 Center for Information Architecture in Research, University of Puerto Rico Medical Campus, San Juan, Puerto Rico, USA; 3 National Center for Biotechnology Information, National Library of Medicine, Bethesda, Maryland, USA

## Abstract

**Background:**

The National Library of Medicine's National Center for Biotechnology Information offers mini-courses which entail applying concepts in biochemistry and genetics to search genomics databases and other information sources. They are highly interactive and involve use of 3D molecular visualization software that can be computationally taxing.

**Methods:**

Methods were devised to offer the courses at a distance so as to provide as much functionality of a computer lab as possible, the venue where they are normally taught. The methods, which can be employed with varied videoconferencing technology and desktop sharing software, were used to deliver mini-courses at a distance in pilot applications where students could see demonstrations by the instructor and the instructor could observe and interact with students working at their remote desktops.

**Results:**

Student ratings of the learning experience and comments to open ended questions were similar to those when the courses are offered face to face. The real time interaction and the instructor's ability to access student desktops from a distance in order to provide individual assistance and feedback were considered invaluable.

**Conclusion:**

The technologies and methods mimic much of the functionality of computer labs and may be usefully applied in any context where content changes frequently, training needs to be offered on complex computer applications at a distance in real time, and where it is necessary for the instructor to monitor students as they work.

## Background

There is a high demand for training in the use of databases supporting resources developed by the National Center for Biotechnology Information (NCBI) [1]. Usually, instructors have to travel to distant sites, or students have to come to the National Library of Medicine (NLM) for this training. It is not always possible for instructors to visit all sites where training is needed, especially if located in other countries. Conversely, travel to NLM can be expensive and inconvenient, particularly for trainees in doctoral or post doctoral programs. Consequently, the application of distant education technologies is an attractive supplement to face to face approaches. Technologies using Internet protocols (IP) are exceptionally useful because the information resources themselves are Internet accessible. In addition, the Internet provides a range of synchronous and asynchronous communication services that can be employed for distant education such as email, the Web, messaging, video streaming (live and on demand), and two way interactive videoconferencing. Given the nature and complexity of the resources taught, it was highly desirable to have the same real time interaction with students at a distance that occurs when teaching is done in person.

The Internet has made it possible to offer more education online and at a distance. This e-learning has taken many forms as the capacity and character of the Internet has changed. The earliest e-learning involved use of email to send information and exercises to students, but soon evolved to using the Web, message boards and streaming video as these additional services came online. Internet protocols are currently robust enough for two way point to point and multipoint videoconferencing, especially over advanced research and education networks such as Internet2 and, to a lesser extent, over the commodity Internet. Although students appreciate the convenience of time and place flexibility asynchronous communication affords in distance education, they prefer classroom instruction and face to face interaction with teachers and peers [2-4]. Consequently, many institutions have adopted blended learning approaches combining face to face and distance learning [5]. Videoconferencing is becoming part of the mix of technologies to provide distance education and blended learning in varied areas of health science [6-9].

Transactional distance, the physical separation that increases the likelihood of miscommunication and misunderstanding in distant education, is a function of teacher-student and student-student interaction [10-12]. Videoconferencing can reduce this distance because it accommodates much of the immediate and non-verbal communication characterizing interaction that is face to face [9,12]. The extent to which synchronous communication via videoconference can substitute for interaction in person is unclear [13,14], but there is evidence that when video is used for teaching, providing bi-directional interaction produces better learning outcomes than when communication is just one way [15] and that student satisfaction levels are higher in distance learning programs when the channel of communication is video versus text [4]. The technology has the potential of extending sense of presence and sense of community, factors having positive affects on student attitudes toward distance learning [16-18]. There is evidence, however, that students at the site where conferences originate interact more than those at distant sites and that instructors concentrate more on students that are physically present [19]. The technology can constrain dialog [16,19] and students at distant sites often feel more disconnected than those at originating ones [17,20]. This detachment can be mitigated when students are not physically present at the origination site so the instructor can concentrate fully on those at a distance [21].

While the ability to see and hear each other and interact in real time is invaluable in a videoconference, a concomitant problem in education involves sharing resources. There not only is a need to engage in conversation, but also to share slides, browsers, and other software to convey information, provide practice, and give feedback and assistance. The ability of teachers and students to interact with the same desktop applications in real time may extend sense of presence and reduce transactional distance by adding a "hands on" dimension to the audio and video communication channels the technology affords.

Since computer applications can be complex and highly interactive, instruction on their use frequently occurs in computer labs. The labs have numerous pedagogical advantages, allowing students to interact instantly with each other and their instructors, have immediate hands on experience performing tasks instructors demonstrate, and complete exercises in which they apply what they have learned to solve more complex problems while the instructor is present to help. Since many of these lab problems, such as formulating and conducting a search, might be solved appropriately in many different ways, it is not always possible to provide pre-packaged feedback about how the problem may be solved or to anticipate and control for varied difficulties students may encounter, an approach common in much asynchronous online education. This problem is compounded in biotechnology because online databases and information sources change continually. The content is constantly updated and features are often added or modified that alter the interface. When instruction is synchronous, it is possible to teach the most up to date version of the application without having to revise online training materials. It is also possible to immediately address variations in retrieval when students employ different search strategies or features of the interface. Instructors can monitor student progress, answer questions, and resolve problems immediately in the laboratory setting. They can walk around the lab, observe students' desktops, and, if the lab is equipped with appropriate switching devices, project any given student's desktop to address issues of interest to the entire class.

NCBI offers several 2.5 hour format mini-courses [22] at the National Institutes of Health and at locations around the country to over 4000 students a year. The courses use a paired problems approach in which the first of two similar problems or problem sets is solved by the instructor during the first hour on a computer linked to a projection system, while the students watch; in the second hour, the students tackle the second paired problem, or set of problems at their own computers, as instructors help the students when needed. These courses have been effective as practical introductions to bioinformatics procedures. The challenge faced in developing the virtual computer lab was how to replicate the desirable features of face to face interaction in actual computer laboratories when education is provided at a distance. Videoconferencing accommodates much of real time interaction and has been implemented at NLM. But there is the additional requirement that faculty and students also share computer applications. Using the Internet to share slides or a web browser on an instructor's desktop with many different students at a distance is routine, but allowing the instructor access to all the distant student desktops to provide assistance in a manner typically done in computer labs is more difficult. Methods were devised to combine bi-directional video and desktop application sharing software to realize some of the benefits of face to face instruction in computer labs at a distance.

## Method

Sharing applications in one direction, from the teacher to the students, can be accomplished several ways. The most widely used technologies for videoconferencing over IP comply with the H.323 standard. This standard embraces several sub-standards for compressing and decompressing video, initiating calls, and sharing data and applications. The H.239 sub-standard has recently been added to its earlier T.120 sub-standard for incorporating desktop applications into a conference. H.239 is used most often with standalone videoconferencing appliances and allows an end point to connect a desktop or laptop to the videoconferencing device so that data from the computer can become part of the video stream. The data consumes most of the screen space and displays at a higher resolution, while video of the presenter can appear in a small window (picture in picture). Prior to implementation of H.239, the data would be displayed as standard NTSC television, without picture in picture capability, and the resolution was often inadequate, especially if presenters wanted to show web pages and other information with smaller text.

H.239 is useful when a single end point (e.g., that of the instructor) needs to display information simultaneously to one or more other end points (e.g., those of the students), but it is insufficient for accommodating sharing from all participants. If an instructor wanted to access the desktops of students at a distant site, each of the student machines would have to be connected to a H.239 device or have H.239 software installed. In addition, the students would need to be familiar enough with its operation to inject display of their desktops into the conference at the appropriate time. Moreover, a multipoint conferencing unit (MCU) is needed that is robust enough to accommodate desktop streams from all participating end points. The MCU and extra individual devices or software add expense. Finally, since end points having older H.323 technology are not able to accommodate the H.239 capability that has been added to the standard, only sites having the latest technology can participate.

The T.120 standard also is used in H.323 videoconferencing products, especially those that can be installed directly on computers. It allows any end point in a conference to share one or more of its applications or its entire desktop with other computers and, optionally, give others control. T.120 functionality is useful, but all participating end points have to initiate sharing with the H.323 videoconferencing software and allow outside control. While an instructor familiar with the product might do this easily to share their presentation, it presumes students are equally familiar with the program to perform the same tasks when they need to show their desktops to receive assistance. In addition, it can add expense if copies of the usually proprietary H.323 software have to be installed on each student machine. Finally, there are technologies for videoconferencing over IP in widespread use that are not based on the H.323 standard and that may lack the functionality of T.120 or H.239.

Video delivered over IP is bandwidth intensive. It consists of a series of individual pictures or frames that are displayed at a rate of thirty per second to create the illusion of smooth motion. This data has to be digitally encoded, transmitted, decoded, and redisplayed at participating sites. Mechanisms for compressing and decompressing video (codecs) are employed to keep the video streams manageable. Videoconferencing technologies can use any number of codecs and the approach to application sharing employed in the virtual computer lab does not rely on using a given codec or videoconferencing approach. This is accomplished by separating application sharing and videoconferencing. Additional software is used that solely performs the application sharing tasks through a second IP connection and that operates concurrently with the videoconferencing program. The students and instructor constantly view each other on the videoconferencing display and the applications they use are seen on a second display. Any commercially available H.323 product (e.g., Polycom, Life Size, or Tandberg), open source program (e.g., Access Grid or Conference XP), or other IP videoconferencing technology can be used to transmit audio and video.

Separating the transmission of audio and video from the transmission of data also adds flexibility in sharing desktops, since there are many desktop sharing software options that can be used. These include relatively recent commercial products, such as Microsoft's LiveMeeting having additional whiteboard, messaging, and other features and Virtual Network Computing (VNC) desktop sharing programs that have been in use for over a decade. The latter are designed especially for sharing applications on remote machines and many of these programs, such as RealVNC and UltraVNC, have versions that can be used for free.

**Figure 1:**
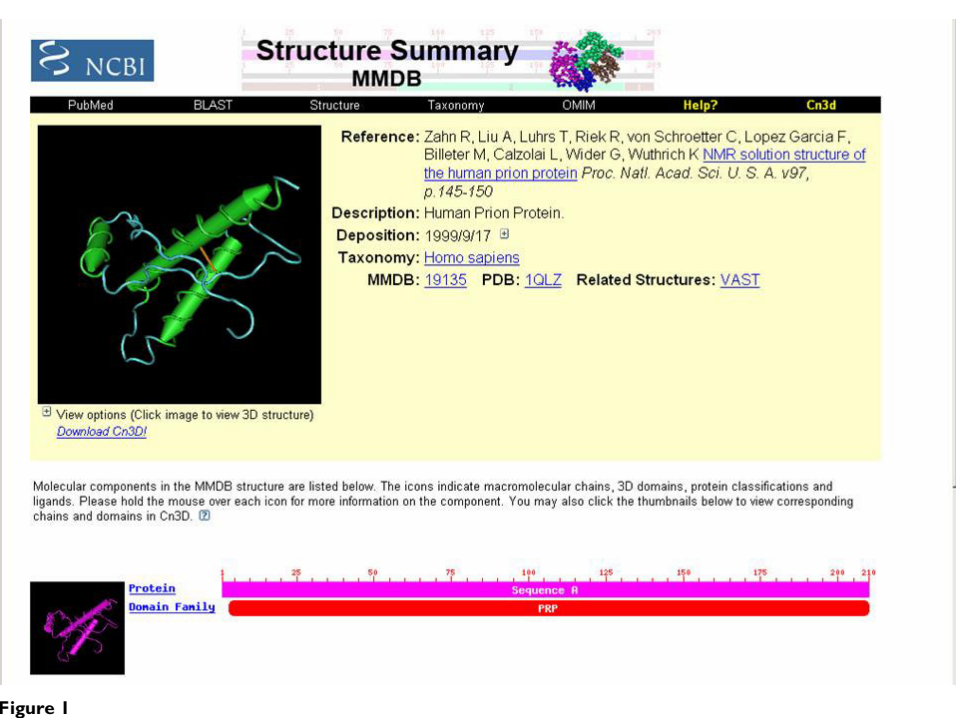
Molecule retrieved from the molecular structure database.

**Figure 2:**
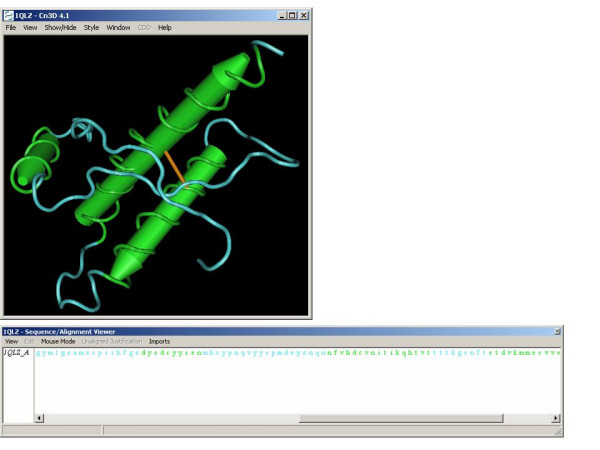
Cn3D three dimensional molecular modeling viewer launched by clicking the image of the molecule retrieved.

In the virtual computer lab, a VNC server runs on any machine that will share its desktop and the machines that will view the desktops run VNC clients and connect to the servers. A particular VNC product was used in the virtual computing lab biotechnology courses only because it enables the smooth manipulation of a NCBI-developed 3D molecular visualization program, Cn3D [23], allowing users to rotate molecules and to highlight, remove, or add certain elements once a molecule is retrieved from a database (Figures 1 and 2). The 32 bit color depth used by the program causes latencies when molecular models are manipulated over networks. The VNC product employed could change the color depth of the shared desktops to 256 colors, reducing latency with minimal degradation in image quality so the models could be manipulated smoothly at a distance. Any desktop sharing program might be used, however, when applications are less computationally intense.

**Figure 3:**
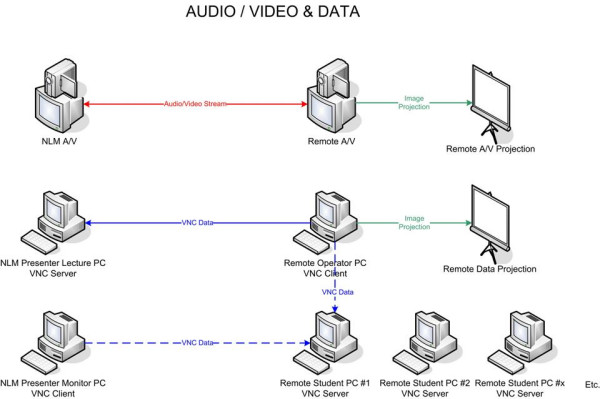
The virtual computer lab connectivity model.

**Figure 4:**
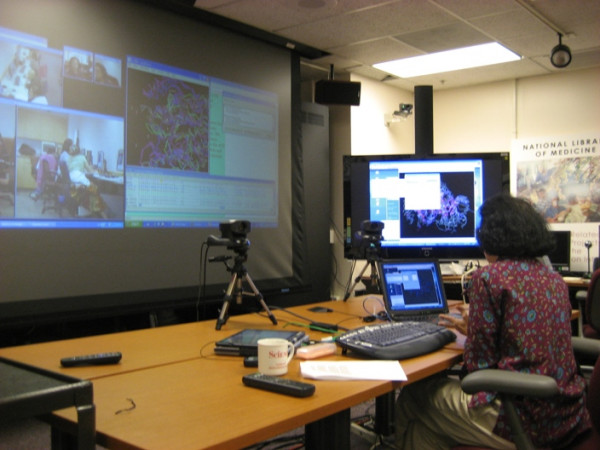
The virtual computer lab in operation at the instructor's site.

In the virtual computer lab, the instructor communicates with the students at a remote computing facility by videoconference and uses a second computer running a VNC server to present the slides, web database interfaces, Java applets and molecular visualization software that are part of the biotechnology education. A computer run by an operator at the distant end point uses a VNC client to connect to the instructor's presentation computer. The operator's machine is projected at the remote site so the class can see the applications the instructor demonstrates. In addition, each student computer is set to run a VNC server transparently in the background prior to each training session. The instructor is able to access the desktops of individual student computers during the hands on sessions that follow demonstrations by using a third computer running a VNC client and preset scripts. The instructor asks the students having problems the name or number of the computer to which they have been assigned and activates the associated script to access and view its desktop. The operator at the remote facility can disconnect from the instructor's machine and run the same preset script to simultaneously access the student desktop and project it (Figures 3 and 4). In this way, the entire class can participate in the review of the problem and the feedback the instructor provides any given student.

## Results

Two pilot implementations of the virtual computer lab were highly successful. The first used H.323 videoconferencing for communication between NLM and the University of Puerto Rico Medical Campus. The second used InSORS, a commercial program based on Access Grid open source videoconferencing, for communication between NLM and the University of Michigan Medical School. The technologies use the same video codecs, but the latter can accommodate simultaneous video streams from multiple cameras. There were no students present at the instructor's site so attention could be devoted entirely to those at a distance in each pilot. In both cases, there were no technical difficulties and the ability to access student desktops proved very beneficial. The instructor could literally diagnose problems the students had at a glance, take control, and fix them.

Eight graduate and post-graduate students from the University of Puerto Rico and seven graduate and post-graduate students from the University of Michigan participated in the two pilot studies and completed the standard evaluation form used for all mini-courses. The form is a five point Likert scale asking students to rate the organization of the lecture and hands on components of the course and the course's usefulness. All the students at the University of Puerto Rico gave the course's organization the highest rating. All ratings but one of the course's usefulness were the highest possible and that rating was second highest. Five of the University of Michigan students gave organization the highest rating and two gave it the second highest. Six rated usefulness highest and one rated it second highest. Overall, the students at both institutions gave the instruction exceptionally high ratings. In addition, students in each pilot had an opportunity to make open ended comments on the evaluation forms. Comments from the students at both schools were highly enthusiastic and the most frequent ones concerned the organization and relevance of the course and the quality of teaching. These evaluation outcomes are similar to those when the course is offered in person, indicating that distance and the technology did not adversely affect how students perceived the learning experience. No data were collected specifically on the two videoconferencing technologies. NLM staff felt both videoconferencing technologies were useful, but that the one used in the second pilot enabling multiple video streams from the remote site provided a better sense of presence, making it easier to see and interact with students.

## Discussion

The virtual computer lab was a viable approach to providing training on complex databases using computationally intense 3D molecular visualization software. There was the high degree of real time discussion and desktop sharing approximating the teaching possible when students and teachers are collocated physically in computer labs. The interaction between sites was in real time with little latency, even when using the molecular visualization software, and there were no technical problems. The lack of technical difficulties was probably due in part to the use of late model computers that could accommodate the modeling software and the use of Internet2, a national high performance research and education network, to communicate between end points. Preliminary tests with the University of West Indies in Jamaica indicate that the virtual computer lab model might be enabled on less advance networks if audio and video is transmitted at lower bandwidth.

The virtual computer lab has two major implementation issues. The first is the need to have an operator at the remote computer facility. Someone has to install the client and server software on the remote machines and create the access scripts, which can be automatically generated by the VNC software with a few keystrokes. The operator has to be present during class sessions to access and project instructor and student machines. The second issue is the VNC desktop sharing software that some consider a security risk. The VNC software can be set to allow showing a desktop while blocking external control. This setting does not impact the use of the instructor's machine but it is undesirable for students, since the instructor often has to take control of their desktops, move windows, and change search strategies when providing students feedback. Security can be raised by instituting passwords to access the VNC servers and the passwords can be incorporated into the scripts automating the access process. If necessary, firewall rules can be adjusted to allow outside access to the student machines only from the IP address of the machine the remote instructor is using.

## Conclusion

The virtual computer lab works over advanced networks and may work over networks having less capacity and quality of service. The methodology is viable in other contexts where computer applications change frequently, immediate interaction is desired, complex technologies are to be learned, and there is a need to monitor student work in real time. In such situations, the time and money required to develop asynchronous learning materials may not be justified, given the quick silver nature of the content. Moreover, it is difficult to engineer such materials when the skills being taught can be applied in varied ways and there many different routes to solving problems. Real time interaction with experts who can diagnose student difficulties on the fly and provide tailored feedback is advantageous in these circumstances.

Real time interaction has the potential of reducing transactional distance and increasing sense of presence in distance learning. In most distant learning contexts, such interaction has been limited to what is written, said, or sometimes shown. The virtual computer lab extends communication at a distance by enabling teachers to directly observe students working with applications and demonstrate performance when providing feedback, much like they would when seated beside the student and temporarily taking control of the mouse and keyboard. The implementation of the virtual computer lab model reported here was undertaken to address a practical need and was assessed using teaching evaluations. Future implementations of the model could more directly assess how extending the kinds of distant interaction impact transactional distance using measures of presence and sense of community as well. NLM will continue to explore using the method in other contexts. There is particular interest in trying the virtual computer lab methodology at other domestic and foreign sites, including those not having access to advanced networks.

## Competing interests

The author(s) declare that they have no competing interests.

## Authors' contributions

CL initiated the distance learning project, collaborated on the instructional and technical requirements, and generated the first draft of the manuscript. AV devised the VNC methodology for accessing student desktops and identified the VNC program meeting display and latency requirements. MB collaborated on the instructional and technical requirements and taught the courses. WL assisted AV in testing VNC programs and approaches. JC collaborated on the instructional and technical requirements and recruited students. All contributed to the further development of the manuscript.

## Pre-publication history

The pre-publication history for this paper can be accessed here:


